# Predicting subcellular location of protein with evolution information and sequence-based deep learning

**DOI:** 10.1186/s12859-021-04404-0

**Published:** 2021-10-22

**Authors:** Zhijun Liao, Gaofeng Pan, Chao Sun, Jijun Tang

**Affiliations:** 1 Department of Biochemistry and Molecular Biology, School of Basic Medical Sciences, Fujian Medical University, 1 Xuefu North Road, University Town, Fuzhou, 350122 FJ China; 2 Department of Computer Science and Engineering, University of South Carolina, 550 Assembly St, Columbia, SC 29208 USA; 3 College of Electrical and Power Engineering, Taiyuan University of Technology, No. 79 Yinze West Street, Taiyuan, 030024 SX China

**Keywords:** Subcellular prediction, Protein sequence, Evolution information, Deep learning, Multiple label classification

## Abstract

**Background:**

Protein subcellular localization prediction plays an important role in biology research. Since traditional methods are laborious and time-consuming, many machine learning-based prediction methods have been proposed. However, most of the proposed methods ignore the evolution information of proteins. In order to improve the prediction accuracy, we present a deep learning-based method to predict protein subcellular locations.

**Results:**

Our method utilizes not only amino acid compositions sequence but also evolution matrices of proteins. Our method uses a bidirectional long short-term memory network that processes the entire protein sequence and a convolutional neural network that extracts features from protein sequences. The position specific scoring matrix is used as a supplement to protein sequences. Our method was trained and tested on two benchmark datasets. The experiment results show that our method yields accurate results on the two datasets with an average precision of 0.7901, ranking loss of 0.0758 and coverage of 1.2848.

**Conclusion:**

The experiment results show that our method outperforms five methods currently available. According to those experiments, we can see that our method is an acceptable alternative to predict protein subcellular location.

**Supplementary Information:**

The online version contains supplementary material available at 10.1186/s12859-021-04404-0.

## Background

Subcellular locations of a protein is crucial in understanding its function and physicochemical characteristics; computational methods are necessary in the research of protein analysis since traditional protein subcellular localization methods are laborious and time-consuming [1]. There are a group of membrane-bound organelles in the eukaryotic cells, such as Nucleus, Centriole, Ribosome, vesicle, Mitochondria, Golgi apparatus and so on [2]. Since such compartments perform various functions decided by protein complexes, ascertain the location of a protein in a cell can direct protein function discovery [3]. Traditional protein subcellular localization methods, such as immunofluorescence techniques and expression system fused with green-fluorescent protein, recognize the subcellular location of proteins from the vision of protein layout which is formed with fluorescent protein and fluorescence microscope [4]. However, those traditional methods are laborious and time-consuming, which become limitations to widely utilize [5]. In order to mitigate those limitations, a lot of computational methods were developed in the past few decades [6].

One major branch of subcellular localization prediction method is to use features extracted from protein sequence and a classifier that separates those features into different patterns to predict the protein subcellular location in cells. The method SubLoc, developed by Hua et al. in 2000, uses basic sequence vector with a dimension of 20 as there are 20 natural amino acids, and Support Vector Machine (SVM) [7] to classify proteins [8]. Each unit of this method’s input vector stands for one amino acid. SVM constructs an optimal hyperplane in the high dimensional feature space to separate samples into positive group and negative group. By using SVM as sample classifier, the protein subcellular localization prediction problem can be solved by finding a suitable kernel function to define the inner product in the feature space.

In order to improve the subcellular localization prediction accuracy, many feature extraction methods based on protein sequence were defined, and many classifiers inherited from SVM were generated. For example, the method published by Shen et al. in 2018 takes multi-kernel SVM as a classifier, and applies features extracted from evolution and physicochemical information other than sequence vectors as the input of classifier [9]. Extra features, such as PsePSSM, PsePP and so on, expand the feature vector of the model to thousands of dimensions, and extend the ability of protein representation that also includes the correlation between amino acid sites that are not adjacent to each other. At the same time, multi-kernel SVM enables the classifier to combine multiple kernel functions that are defined for different feature spaces into one kernel function with suitable weights [10, 11]. To improve the proficiency, Ding et al. applied Fuzzy Support Vector Machine (FSVM) instead of multi-kernel SVM in the classification task. Fuzzy support vector machine was developed by Lin et al. based on standard SVM with an extra value for each sample to denote its membership value, with a goal of adjusting the weights of outliers in the trained classification model [12, 13]. Ding et al. applied KNR (Kernelized Neighborhood Representation) function, a kernel function proposed by them to identify protein crystallization that generates the membership values from input samples for FSVM [14], to fully represent the spatial distribution of feature space and control the interference of outliers effectively [15].

Features of proteins can be extracted not only from raw protein sequences but also from correlations between amino acid sites indirectly. The method proposed by He et al., Imbalanced Multi-Model Multi-Label Learning with Gaussian Process (IMMMLGP), is one of the methods that uses sequence information and Gaussian Process Prior to predict subcellular localization of protein [16]. The authors of this method applied a gaussian process model to effectively exploit the correlations among multiple locations that are important in improving the prediction accuracy of multi-localized proteins [17, 18]. Furthermore, this method is robust on imbalanced data sets because optimal linear combinations of various feature extraction technologies were included. Currently, a Java library named MULAN, which was proposed by Tsoumakas et al., offers programmatic application interface for multi-label learning [19]. Wei et al. selected 11 simple multi-label classifiers from MULAN library to fuse a mean ensemble classifier after each single classifier was trained and clustered [20]. Overall, the general procedure of building up a subcellular localization prediction method is to first extract features from protein sequence comprehensively; then find a suitable classifier algorithm according to the feature space and adjust the classifier to fit the problem; lastly analyze the performance of the classifier after training and testing on some benchmark datasets.

The prediction method mGOASVM utilizes BLAST [21] search to find out the accession numbers of a given protein’s homologs, then it searches the Gene Ontology annotation database for the GO terms of the given protein. The GO terms are processed into feature vectors, then those features are used to train a SVM classifier which can predict the subcellular locations of protein accurately. In addition to the achievement of mGOASVM, the prediction method mLASSO-Hum takes one step further. It uses a one-vs-rest LASSO-based classifier, instead of a SVM classifier to improve the interpretability of the classification results and the robustness of classification model [22]. Since the GO terms have high dimensional features, classifiers using this kind of information tend to overfit the train dataset. The LASSO-based classifier identified important features from all the GO terms, by which the dimension of the feature vector was significantly reduced. With these advantages introduced by informative GO terms and functional classifiers, the method mLASSO-Hum outperforms several other subcellular localization methods on prediction accuracy. Furthermore, its result has strong interpretability which provides insight into the relationship between proteins and subcellular locations [23].

Protein sequence information and its GO annotations are widely used in subcellular location prediction methods. For example, the method Hum-mPLoc 2.0, proposed by Shen et al. in 2009 established on Hum-mPLoc, is a synthesized prediction method that incorporates both protein sequence and GO terms [24, 25]. The method Hum-mPLoc takes the GO terms into account but is still restricted by the shortage of accession numbers and evolution information, so the performance of this predictor is not as satisfactory on some specific problem sets. For this reason, Shen et al. made improvements on this method by adding domain and evolution information extracted from protein sequence to the feature list, which boosted the performance of the classifier on the problem sets [26]. The method iLoc-Hum is another method that uses GO terms besides protein sequence [27]. Prediction methods, such as pLoc_bal-mAnimal [28] and pLoc-mGneg [29], apply over-sampling algorithm and under-sampling algorithm on imbalanced datasets to balance the training dataset, and train the prediction model with balanced samples in order to overcome the limitation of machine learning algorithm.

Deep neural networks extract features from input data automatically and predict the output of the model based on those features, which can predict the subcellular locations of a protein sequence without feature extraction methods. Deep learning has become more and more popular in the research field of artificial intelligence in the past few years [30]. There are a lot of deep neural network models that focus on accurate prediction of protein subcellular localization. The method DeepLoc, proposed by Armenteros et al. in 2017, takes recurrent neural network to processes the entire protein sequences, and it also applies attention mechanism which identifies the important protein regions for the subcellular localization in the neural network model [31]. With the support of the recurrent neural network, feature extraction methods are omitted from the construction of prediction method. And the attention mechanism improves the accuracy of prediction.

Before training the neural networks, protein sequences can be reformulated to generate an effective representation of the biological sequence samples. Sequence based subcellular localization prediction methods, such as pLoc_Deep-mHum [32], pLoc_Deep-mEuk [33], pLoc_Deep-mvirus [34] and so on, pre-process the protein sequences with Pseudo Amino Acid Composition algorithm [35], then train a convolutional neural network or a recurrent neural network with the new generated property vectors. The neural network can learn the patterns in proteins more effectively from the reformulated properties than from the original protein sequences. The method DeepPSL, developed by Wei et al., uses an adaptive skip dipeptide composition to calculate the fraction of adjacent residues in the protein sequences, then uses a stacked auto-encoder to encode those fraction properties into subcellular location labels [36]. Protein physicochemial properties are also used by DeepPSL as a sequence representation; those properties enlarge the feature space and improve the prediction accuracy [37].

Deep learning models can predict protein subcellular locations from images. The method ImPLoc, proposed by Long et al., predicts protein subcellular locations from IHC (immunohistochemistry) images [38]. In this method, deep convolutional neural network extracts image features, self-attention encoder aggregates the extracted feature vectors, fully connected network predicts the location labels. Similar to ImPLoc, the method DeepYeast also applies deep convolutional neural network in the subcellular localization prediction from high throughput microscopy images [39]. Image pre-processing algorithms, such as Gamma correction, Morphological Closing and so on, are widely used to improve the quality of images. The method proposed by Masurkar et al. takes this strategy to revise the result predicted from microscopy images [40]. Deep learning was also applied in the prediction of messenger RNA subcellular location or long non-coding RNA subcellular location. Those RNA subcellular localization prediction methods include IncLocator [41], RNATracker [42], DeepLncRNA [43] and so on.

In this paper, we introduce a deep learning-based protein subcellular localization prediction method which uses bidirectional long short-term memory network as an encoder and takes evolution information into account. Experiment results shows that our method can predict protein locations accurately.

## Method

### Protein sequence encoding

Amino acids construct various proteins that can fulfill the basic functions in cells. Each kind of amino acid has a unique side chain that determines its property and action. The amino acid sequence represents the primary structure of a protein. The composition of amino acids decides the structure and function of proteins, so the amino acid sequence becomes the major research target to analyze proteins. Given a protein sequence *P* which consists of *n* amino acids, it can be defined as:

$$p=[a_0, a_1, a_2, a_3, \ldots , a_n]$$

and $a_i$ represents the amino acid at *i*th position in the protein.

One of the most widely used encoding algorithm in computational biology methods is one-hot encoding, which converts nucleotide sequence or amino acid sequence into a list of binary vectors [44, 45]. Each amino acid will be encoded into a binary vector with only one specific position being 1 and other positions being 0s based on its type. The one-hot encoding of protein *p* is:

$$P = \left[\begin{array}{ll} p_{0, 0}, p_{0, 1}, p_{0,2}, \ldots , p_{0, 22} \\ p_{1, 0}, p_{1, 1}, p_{1,2}, \ldots , p_{1, 22} \\ \vdots \\ p_{n, 0}, p_{n, 1}, p_{n,2}, \ldots , p_{n, 22} \\ \end{array}\right]$$

in which $p_{i,j}$ is 0 or 1, and $\sum _{j=0}^{22}p_{i,j} = 1$. The encoded matrix can be used to train neural network.

### Position specific scoring matrix

Position Specific Scoring Matrix (PSSM), introduced by Gary Stormo et al. in 1982, is the consensus score and evolution information of protein sequences [46, 47]. The score in the matrix is the substitution probability of amino acids at specific positions in a protein sequence. High score means more frequent substitution in an alignment, which low value means less frequency.

PSSM can be gnerated by BLASTing the protein sequence with Position Specific Iterated BLAST (PSI-BLAST) tool on the server of National Institutes of Health, of which the URL is: https://blast.ncbi.nlm.nih.gov/Blast.cgi?PROGRAM=blastp.

The PSSM of a protein sequence *p* is:

$$S = \left[\begin{array}{ll} s_{0, 0}, s_{0, 1}, s_{0,2}, \ldots , s_{0, 42} \\ s_{1, 0}, s_{1, 1}, s_{1,2}, \ldots , s_{1, 42} \\ \vdots \\ s_{n, 0}, s_{n, 1}, s_{n,2}, \ldots , s_{n, 42} \\ \end{array}\right]$$

in which $s_{i,j}$ is the substitution frequency from *i*th amino acid in the protein sequence to amino acid *j*. Positive values indicate the substitution occurs more frequently, while negative values indicate less frequent substitution. A convolutional neural network extracts features from the generated PSSM. Those features represent the consensus properties of protein functions.

The protein sequences used in the experiment of this project had been pre-processed and the corresponding PSSM was uploaded into github repository (https://github.com/Paeans/subcellular).

### Bidirectional long short-term memory network

Long short-term memory (LSTM) network is a special kind of recurrent neural network (RNN), which is widely used in the field of deep learning to process time series data [48]. The LSTM network has four interactive neural network layers which combine the previous states and current input. The architecture of LSTM avoid the long term dependency problem, and the output formulate the entire sequence of input data [49]. Bidirectional LSTM (BLSTM) contains two LSTM networks, of which one LSTM network process protein sequence from beginning to end while another one process the sequence from end to beginning [50]. Since the LSTM network can memory the states at different locations of a sequence, it was widely used to encode sequence samples [51].

With a one hot encoding matrix of a protein *P*, the LSTM network can encode the matrix into a state matrix which includes the functions expressed by different protein sites. For an input $P_t$, the state and output of the model node are defined as:

$$\begin{aligned} f_t &= \sigma (W_f \times [h_{t_1}, P_t] + b_f) \\ i_t &= \sigma (W_i \times [h_{t-1}, P_t] + b_i) \\ \tilde{C_t}& = tanh(W_C \times [h_{t-1}, P_t] + b_C) \\ C_t& = f_t \times C_{t-1} + i_t \times \tilde{C_t} \\ h_t& = \sigma (W_h \times [h_{t-1}, P_t] + b_h) \times tanh(C_t) \end{aligned}$$

and $W_f$, $W_i$, $W_C$ and $W_h$ are the weight matrices applied on input matrix, $b_f$, $b_i$, $b_C$ and $b_h$ are the bias matrices, $f_t$ is the forgetting coefficient, $i_t$ is the remembering coefficient, $C_t$ is the current state and $h_t$ is the output.

The Bidirectional LSTM network processes the protein sequence from two directions. The raw vector of amino acids is translated into another numerical vector which includes position information and relation information between different function sites.

### Convolutional neural network

Convolutional neural network (ConvNet) utilizes regularized filters and activation nodes to discover patterns from input matrices; it had been widely used in image processing, natural language processing, financial trends analysis, and so on [52–55]. ConvNet also has a lot of applications in the research fields of computational biology, such as non-coding gene function prediction [56], methylation sites classification on whole genome sequence [57], protein-protein interaction analysis [58], and low quality Hi-C data denoising [59]. ConvNet can extract features from raw input automatically, and the important features can be filtered out with kernel weights.

**Fig. 1:**
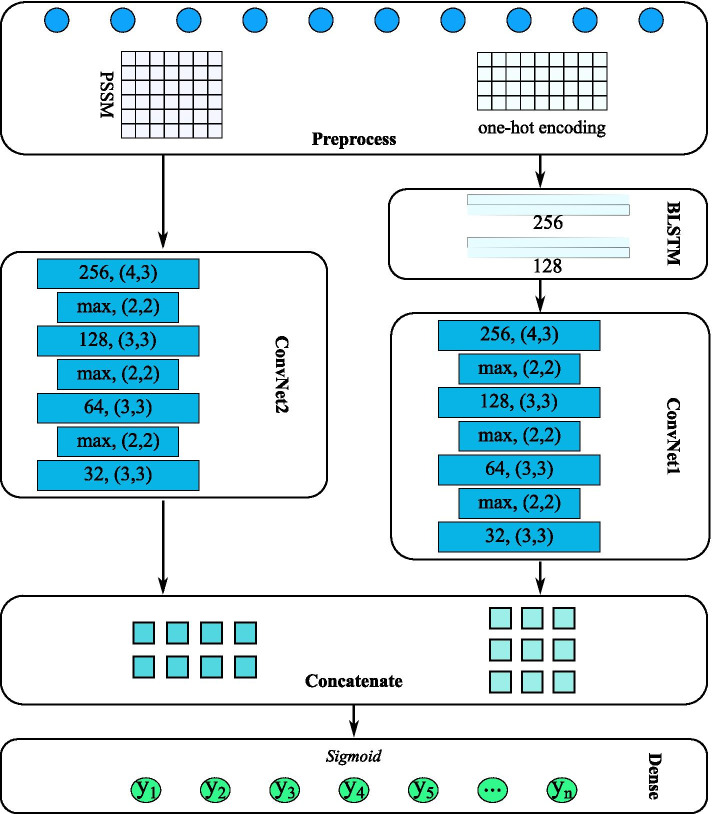
The workflow of our method. Our method composes 5 components which are sequence pre-process layer, bidirectional LSTM encoder, 2 convolutional neural network and prediction layer. **a** Sequence pre-process layer processes protein sequences into one-hot encoding matrix and position-specific scoring matrix. One protein sequence is converted into two matrix which are the inputs of following deep learning neural networks. **b** Bidirectional LSTM encoder takes an one-hot encoding matrix as input and processes the items in the matrix sequentially. This encoder includes two LSTM layers. One layer processes the matrix from beginning to end while another layer processes the matrix backwards from end to beginning. After two direction encoding, the original one-hot encoding matrix is encoded to 256 values. **c** Convolutional neural network. The convolutional neural network is used to extract features from pssm matrix and encoded matrix. Two identical neural networks are used in our method. One network learns pssm matrix and another one learns encoded matrix. In the network, 4 convolution layers are included to filter out main features and 3 maxpooling layers are inserted among the convolution layers to choose outstanding features. The number of kernels in the 4 convolution layers are 256, 128, 64 and 32 respectively, and the kernel size of each layer is 4 × 3, 3 × 3, 3 × 3 and 3 × 3 correspondingly. d). Prediction layer produces the possibility of each subcellular location. At the beginning of this layer, the outputs from previous two convolutional neural networks are concatenated together, then the concatenated matrix is flattened into one dimensional array. A fully connected network integrate those features together and Sigmoid function computes the corresponding possibility at each location. Based on the output possibility, the subcellular locations of a protein can be decided

The convolutional layers are the core building blocks of ConvNet; those layers consist of a group of kernels that can be adjusted according to the loss values of prediction during the backward pass [60]. The kernels in ConvNet are small size matrices. The convolution function between kernel matrix $\omega$ and input volume *X* is:

$$C_{i,j} = \sum _{m,n = 0}^{|\omega |} \omega _{m,n}X_{i + m, j + n}$$

and $C_{i,j}$ is the convolution value of kernel $\omega$ on region $X_{[i:i+m, j:j+n]}$. With backpropagation algorithm, the values in kernel $\omega$ can be optimized according to the value $\frac{\partial L}{\partial \omega }$ which is the partial derivative of error *L* with respect to $\omega$ [30]. Figure 1 shows the architecture of our method.

### Activation function

Activation function transforms the weighted input into an activation output of the neural network node. This function simulates the stimulation happened between biological neurons [61]. In this method, rectified linear activation function (ReLU) is applied in the convolutional layer nodes and sigmoid function is used to generate output labels. The ReLU in the node is defined as:

$$r(x) = max(0, x)$$

which means the node can only be stimulated by signals that are strong enough [62]. It is a linear function when the input is greater than 0, but it also introduces non-linearity property into the neural network model at point 0 [63].

Sigmoid function translates the input into a value in the range 0 to 1. The formula of this function is:

$$S(x) = \frac{e^x}{e^x + 1}$$

with *x* as the input of node. When the input signal is strong, the output possibility value is close to 1, otherwise the output value is close to 0. On multi-label classification problems, the sigmoid function is used in the output layer of the neural network to generate the possibility for each output label [64].

### Loss function

Loss function maps prediction values onto real values representing the distance between model outputs and targets. The optimization algorithms minimize the loss function during training process. Protein subcellular localization prediction method needs to generate multiple binary labels for a protein; in order to train the model in our method effectively, binary cross entropy loss function is applied to compute the cross-entropy loss between predicted labels and true labels [65].

*L*2 regularization prevents model overfitting by imposing a cost on the loss function [66]. The *L*2 penalty $||\omega ||_2^2$ is the squared magnitude of kernel in ConvNet, and this penalty can regulate the weights applied on input signals [67]. The loss function with *L*2 regularization is:

$$L = -\frac{1}{n} \sum _{i=1}^{n} \big ( y_i \times log\tilde{y_i} + (1-y_i)\times log(1-\tilde{y_i}) \big ) + \lambda ||\omega ||_2^2$$

in which $\tilde{y_i}$ is the prediction on *i*th label while $y_i$ is label *i* with value 0 or 1, *n* is the size of labels, $\omega$ is the kernel of ConvNet while $\lambda$ is the coefficient of regularization penalty.

## Results

### Benchmark datasets

Our method was tested on two benchmark datasets, *D*3106 and *D*4802. Benchmark dataset *D*3106, generated by Shen et al. in 2009, includes 3106 protein sequences which are located at 14 subcellular locations [26]. The number of protein sequences located at each subcellular location in dataset *D*3106 is listed in Table 1 and the detailed information about correlations of subcellular locations is listed in Additional file 2. Benchmark dataset *D*4802, generated by Wei et al. in 2016, contains 4802 protein sequences located at 33 subcellular locations [20]. The number of proteins at each subcellular location in dataset *D*4802 is listed in Table 2 and the correlations between each subcellular location are listed in Additional file 3. The protein sequence distribution in Tables 1 and 2 shows that most of the proteins are located in Nucleus and Cytoplasm, and only a small number of proteins are in Peroxisome and Synapse; the samples in the datasets are unbalanced.

**Table.1:** Number of protein sequences in benchmark dataset *D*3106 created by Shen et al.

Subcellular location	Number of protein sequences
Nucleus	1021
Cytoplasm	817
Extracellular	385
Mitochondrion	364
Plasma membrane	354
Endoplasmic reticulum	229
Golgi apparatus	161
Cytoskeleton	79
Centriole	77
Lysosome	77
Peroxisome	47
Endosome	24
Microsome	24
Synapse	22
Total	3681

The dataset *D*3106 covers 14 subcellular which are listed at the first column of this table. And the numbers of proteins located at each subcellular location are listed at the second column. There are 3106 protein sequences in this dataset, and the total number of subcellular locations is 3681 since many certain sequences can be found in multiple locations. The sequences distribute at those 14 locations unevenly. 32.9% sequences are located at Nucleus and 26.3% sequences are at Cytoplasm, while less than 1% sequences are located at Synapse. This dataset is unbalanced. 3681 positive cases take only 8.47% of all 3106 × 14 cases

The correlations between each subcellular location of dataset D3106 which are listed in Additional file 2 respectively show that there are 480 proteins in D3106 which are located in two different subcellular, while 43 proteins exist at three different locations and 3 proteins are in four cell constituents at the same time. Dataset D4802 has 1354 two-subcellular samples, and the number of those protein samples is in Additional file 3. From the table in Additional file 3, we can see that 401 protein sequences exist at Nucleus and Cytoplasm at the same time and 268 protein sequences exist at both Nucleus and Nucleolus, between which high correlations are exhibited.

### Evaluation metrics

To evaluate the accuracy of prediction results, ranking loss, coverage and average precision are commonly used in multi-label deep learning [68, 69]. Those functions are defined as:

ranking loss

$$RL = \frac{1}{n} \sum _{i=0}^{n-1} \frac{|\{(k, l): \tilde{y_{ik} } \le \tilde{y_{il}}, y_{ik} = 1, y_{il} = 0 \}|}{||y_i||_0(n - ||y_i||_0)}$$

coverage

$$Cov = \frac{1}{n} \sum _{i=0}^{n-1} \max _{j:y_{ij} = 1} |\{k: \tilde{y_{ik}} \ge \tilde{y_{ij}}\}|$$

average precision

$$AP = \frac{1}{n} \sum _{i=1}^{n-1} \frac{1}{||y_i||_0}\sum _{j:y_{ij} = 1} \frac{|\{k: y_{ik} = 1, \tilde{y_{ik}} \ge \tilde{y_{ij}}\}|}{|{k: \tilde{y_{ik}} \ge \tilde{y_{ij}}}|}$$

in those functions, *n* is the number of samples, $y_{ij}$ is the ground truth label of sample *i* at label *j*, and ${\tilde{y}}$ is the output score matrix of all the samples. |*X*| is the number of elements in the set *X* while $||X||_0$ is the number of nonzero elements in the set. Those three functions are defined based on the label pairs that are incorrectly ordered. For example, ranking loss function computes the proportion of true labels which get lower score than false labels, coverage function computes the average rank of lowest prediction values for true labels, and average precision is the average fraction of true labels among all the labels which are higher-ranked than the lowest true label rank. Those three functions will be used to evaluate the performance of our method on the test samples.

The F1 score (*F*_1_), Matthews Correlation Coefficient (MCC) and Area under the ROC curve (AUC) are defined to measure the quality of binary classification [70]. The balanced F1 score in machine learning can measure the quality of binary classifications on unbalanced datasets. It is a weighted average of the precision and recall with values range from 0 to 1. The function of F1 score is:

$$F_1 = 2\cdot \frac{PPV\cdot TPR}{PPV + TPR}$$

with precision *PPV* = *tp*/(*tp* + *fp*) and recall *TPR* = *tp*/(*tp* + *fn*) in which *tp*, *tn*, *fp* and *fn* are true positive, true negative, false positive and false negative, respectively.

The MCC is a balanced measure of the quality of binary classifications even when the test dataset is an unbalanced dataset [71]. The value of MCC is between $-1$ an inverse prediction and 1 a perfect prediction. The MCC function for binary classification is:

$$MCC = \frac{tp \times tn - fp \times fn}{\sqrt{(tp + fp)(tp+fn)(tn+fp)(tn+fn)}}$$

and *tp*, *tn*, *fp* and *fn* are the true positive, true negative, false positive and false negative, respectively.

The AUC computes the area under the receiver operating characteristic (ROC) curve, which is created with true positive rate and false positive rate at various threshold [72]. It reflects the probability that a classifier generates higher scores on true instances than on false instances. We will compute the F1 score, MCC and AUC on each subcellular label to evaluate the performance of our method.

### Model performance

In this experiment, we test four different models with benchmark datasets D3106 and D4802 and compare the prediction accuracy of those models with five existing subcellular localization methods. The four models tested in this experiment are model BLSTM which uses 2 bidirectional LSTM layers to encode the protein sequences into subcellular location labels, model BLSTM plus ConvNet1 which takes 4 convolution layers and 3 maxpooling layers following 2 bidirectional LSTM layers, model ConvNet2 which utilizes PSSM of protein sequences and a 7 layer convolutional neural network to predict protein subcellular locations, and model BLSTM plus 2 ConvNet which uses BLSTM, ConvNet1 and ConvNet2. At first, we evaluate the performance of each model on single subcellular location prediction, then evaluate the accuracy of multi-subcellular locations prediction. The detailed testing results can be found in additional file document.

**Table.2:** Number of protein sequences in benchmark dataset 4802 created by Wei et al.

Subcellular location	Number of protein sequences
Nucleus	1720
Cytoplasm	1050
Plasma membrane	836
Extracellular	487
Mitochondria	407
Endosome	342
Golgi apparatus	272
Nucleolus	268
Lysosomes	125
Endoplasmic reticulum	120
Cytoskeleton	89
Centrosome	81
Peroxisome	67
Early endosomes	52
Nuclear envelope	47
Cytoplasmic vesicles	46
Basolateral plasma membrane	29
Synaptic vesicles	28
Microtubule	26
Apical plasma membrane	16
Late endosomes	16
Golgi trans face	11
Secretory granule	10
Tight junction	9
Golgi cis cisterna	7
Medial-golgi	7
Melanosome	6
Secretory vesicles	5
Cellular component	4
ERGIC	4
Inner mitochondrial membrane	4
Transport vesicle	4
Golgi trans cisterna	3
Total	6198

In this dataset, 4802 protein sequences are identified in 33 subcellular locations. The first column is the name of the subcellular covered by this dataset, and the second column is the number of proteins located at each subcellular location. The total number of subcellular locations is 6198 since each sequence can be found in multiple subcellular locations. The sequences distribute at those 33 locations unevenly. 35.8% of sequence samples are located at Nucleus and 21.9% sequences are located in Cytoplasm, while only 3 sequences are identified in Golgi Trans Cisterna. The number of positive cases in this dataset is 6198, and the positive case rate is 3.9% (6198/(4802 × 33))

The performance of the four models on single label prediction is listed in Table 3. On dataset D3106, the AUC of model BLSTM reaches to 0.9242 on subcellular Lysosome, but the worst AUC is 0.8315 on subcellular Peroxisome. The average AUC of this model on dataset D3106 is 0.8841. The model BLSTM performs better on dataset D3106 than on dataset D4802, while the best AUC on D4802 is 0.9121 which is worse than the best result on D3106 and the average AUC on D4802 is 0.1178 less than the average result on D3106. The values of MCC and *F*1 score also show that model BLSTM works better on dataset D3106.

Full list of AUC values of each subcellular location prediction on dataset *D*3106 is in Additional file 4 and statistical values are listed in Additional file 1: Table S1. The statistical values in Additional file 1: Table S1 show that the model BLSTM plus 2 ConvNet has best performance on 10 of 14 subcellular location predictions, while the model BLSTM plus ConvNet1 performs best on other 4 predictions. Predictions on 8 subcellular locations have average AUC values greater than 0.9 and the standard deviations of those predictions are less than 0.03 on AUC values. On dataset *D*4802, all AUC values of prediction are in Additional file 5 and statistical values are listed in Additional file 1: Table S2. Additional file 1: Table S2 shows that the model BLSTM plus ConvNet1 is the best model to prediction subcellular locations based on samples in dataset *D*4802 and the model BLSTM plus 2 ConvNet can not out perform it. Model BLSTM plus ConvNet1 gets highest AUC on 30 subcellular location predictions and most of those 30 predictions have AUC value greater than 0.9. However, the standard deviation of predictions by model BLSTM plus 2 ConvNet is lower than the value of predictions by model BLSTM plus ConvNet1. The standard deviation reduction shows that the extra ConvNet2 makes the model more stable.

**Table 3:** The *F*_1_ score, MCC and AUC of subcellular location prediction generated by model BLSTM, BLSTM + ConvNet1, ConvNet2 and BLSTM + ConvNet1 + ConvNet2

	***D*3106**			***D*4802**		
	***F***_**1**_	***MCC***	***AUC***	***F***_1_	***MCC***	***AUC***
BLSTM	0.7473	0.6001	0.9242	0.7419	0.5705	0.9121
BLSTM + ConvNet1	0.7775	**0.6419**	0.9255	0.7801	0.6284	0.9327
ConvNet2	0.6475	0.4819	0.8785	0.6696	0.4259	0.9297
BLSTM + ConvNet1 + ConvNet2	**0.7843**	0.6410	**0.9458**	**0.7842**	**0.6411**	**0.9434**

The four models were tested on datasets *D*3106 and *D*4802. On dataset *D*3106, the highest *F*_1_ score and AUC are achieved by the model BLSTM + ConvNet1 + ConvNet2, while the model BLSTM + ConvNet1 has the highest MCC. On dataset *D*4802, the model BLSTM + ConvNet1 + ConvNet2 was the best among the four models

The model BLSTM plus ConvNet1 achieves similar performance on dataset D3106 as the model BLSTM with best AUC 0.9255, MCC 0.6419 and *F*1 score 0.7775. However, the best AUC of model BLSTM plus ConvNet1 on D4802, 0.9327 is better than the achievement on D3106, which is different from model BLSTM. The *F*1 score of model BLSTM plus ConvNet1 on D4802 is 0.7801, and the MCC is 0.6284. Overall, the model BLSTM plus ConvNet1 has better performance than model BLSTM. So, we can see that the ConvNet1 takes effects during the learning process, since the convolution layers can extract features from the encoded vectors. The performance of our method on single subcellular location prediction shows that BLSTM component in our method is the most important part to make accurate prediction on protein subcellular localization. In our method, BLSTM component encodes protein sequence into feature matrices. So the key factor of subcellular localization model is encoding method. This encoding method can also be applied to other biological computational methods, especially methods which take protein sequences as input.

Model ConvNet2 with PSSM as inputs is out of expectations. On dataset D3106, model ConvNet2 only gets 0.8785 of AUC, 0.4819 of MCC and 0.6475 of *F*1 score. On dataset *D*4802, the best MCC of this model is .4259 which is worse than the performance of this model on dataset *D*3106. When comparing the testing results of this model and BLSTM model on datasets *D*3106 and *D*4802, we can see that BLSTM model performs better than this model. The reason of bad performance is that the PSSM can represent the evolution information of proteins but can not replace the sequence information in subcellular location prediction. With bidirectional LSTM, protein sequences can be translated into matrix form which is rich of function information.

When appending ConvNet2 to the model BLSTM plus ConvNet, the accuracy of single subcellular location prediction has no big difference. The model BLSTM plus 2 ConvNet gets best AUC on most of the 14 subcellular locations in dataset *D*3106 according to the results listed in Additional file 1: Table S1. However, when using t test to evaluate the difference between models with and without ConvNet2, we can see that the difference is not significant to prove that ConvNet2 makes the model better on single subcellular prediction ($p_{value} > 0.5$). The t test results are listed in Additional file 1: Table S3. Testing results show similar conclusion with dataset *D*4802. The AUC of each subcellular location prediction has no significant changes when ConvNet2 was appended to the model BLSTM plus ConvNet. So, ConvNet2 has no effect to single subcellular prediction. The statistical values in Additional file 1: Table S3 show that ConvNet1 makes great improvement to the accuracy of single subcellular location prediction on both dataset *D*3106 and dataset *D*4802.

**Table 4:** The average precision, ranking loss and coverage of model BLSTM, BLSTM + ConvNet1, ConvNet2 and BLSTM + ConvNet1 + ConvNet2

	***D*3106**			***D*4802**		
	***RL***	***Cov***	***AP***	***RL***	***Cov***	***AP***
BLSTM	0.0967	1.5895	0.7523	0.0820	3.3916	0.6901
BLSTM + ConvNet1	0.0778	1.3113	0.7876	**0.0603**	**2.9225**	**0.7453**
ConvNet2	0.1294	2.0113	0.6430	0.0673	3.2868	0.6214
BLSTM + ConvNet1 + ConvNet2	**0.0758**	**1.2848**	**0.7901**	0.0637	3.0528	0.7414

On dataset D3106, the BLSTM + ConvNet1 + ConvNet2 has the best performance with lowest ranking loss, coverage and highest average precision which are 0.0758, 1.2848 and 0.7901, respectively. However, the model BLSTM + ConvNet1 + ConvNet2 is not as good as model BLSTM + ConvNet1 when tested on dataset *D*4802. The best values are marked out with bold text

**Fig. 2:**
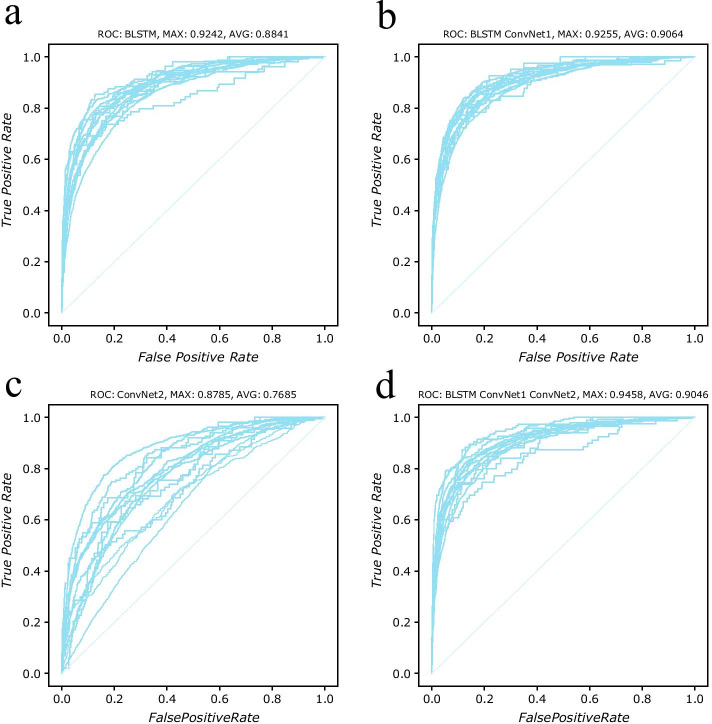
ROC curves (Receiver operating characteristic curve) of model BLSTM, BLSTM + ConvNet1, ConvNet2 and BLSTM + ConvNet1 + ConvNet2, on benchmark dataset *D*3106. Those models were tested with fivefold cross validation. This figure plots the result of one fold validation, and all five fold testing results can be found at Additional file 1: Table S3 and Additional file 1: Figure S1. **a** 14 curves of model BLSTM. The best one with AUC value 0.9242 is achieved on location prediction of subcellular Lysosome, while prediction of subcellular Peroxisome is the worst one with AUC value 0.8480. The average AUC value of this model is 0.8841. The plot shows that the curves of predictions on 13 subcellular locations, except subcellular Peroxisome, are centered together. **b** Validation of model BLSTM + ConvNet1. With an extra convolutional neural network to extract features from encoded protein sequences, the AUC on subcellular Lysosome and Peroxisome reached to 0.9255 and 0.9134. The average AUC value of this model is 0.9064. All 14 curves are convergent together. This model is the most robust model among all the four models. **c** ROC curves of model ConvNet2. Different from curves in (**b**), those 14 curves are divergent. The max AUC value is 0.8785 and the min AUC value is 0.7058. Since it only extracts features from evolution information, this model performance isn’t as good as the other three models. **d** With combination of sequence information and evolution information, model BLSTM + ConvNet1 + ConvNet2 improves the best AUC value to 0.9458. However, predictions of several subcellular locations are effected by the parameters in Convnet2, so the curves of those predictions are divergent from center

**Fig. 3:**
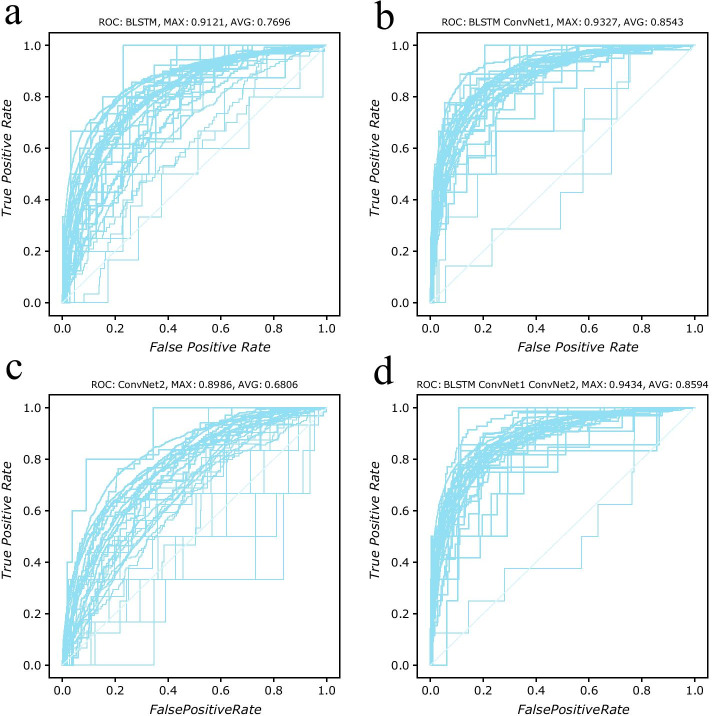
ROC curves (Receiver operating characteristic curve) of model BLSTM, BLSTM + ConvNet1, ConvNet2 and BLSTM + ConvNet1 + ConvNet2, on benchmark dataset *D*4802. This figure plots one fold validation result. Detailed information about the fivefold cross validation results can be found at Additional file 1: Table S4 and Figure S2. Those plots shows that those four models perform worse on dataset *D*4802 than on dataset *D*3106. **a** 33 curves of model BLSTM. The best AUC is 0.9121, however the worst one is 0.7072. The average AUC value of this model is 0.7696. The plot shows that a small group of curves are centered together, while other curves are divergent from one another. **b** 33 curves of model BLSTM + ConvNet1. Two curves around the diagonal show that the accuracy of prediction on two subcellular positions is bad. The average AUC value of this model is 0.8543 which is better than the average value of model BLSTM. **c** ROC curves of model ConvNet2. This plot is similar to the (**c**) in Figure2 but with several curves under diagonal. So the prediction on some subcellular locations isn’t better than random guess. The max AUC value is 0.8986 and the min value is 0.5080. The average AUC value is 0.6806. **d** In this plot, there is still one curve that is under diagonal. However, the best AUC value reaches to 0.9434 and the average AUC value reaches to 08594. In general this model performs better than the other three models

The best AUC and average AUC of the joined model on dataset D3106 are 0.9458 and 0.9046, respectively. According to the AUC values, the joined model has great improvements on protein subcellular localization prediction. The *F*1 score and MCC of this model, 0.7843 and 0.6410 respectively, are also higher than the values of another three models. We plot the ROC curves of predictions in Figs. 2 and 3. All the ROC curves of fivefold cross validation are plotted in Additional file 1: Figures S1 and S2. The plots in supplement figures are similar to the corresponding ones in Figs. 2 and 3 since each model has similar performance on each fold data. Those plots show that model BLSTM plus ConvNet1 is better than the joined model since some predictions created by joined model are not good enough. The reason of accuracy deduction is that the ConvNet2 network introduces extra parameters into the joint model and those parameters affect the training process and model optimization.

**Fig. 4:**
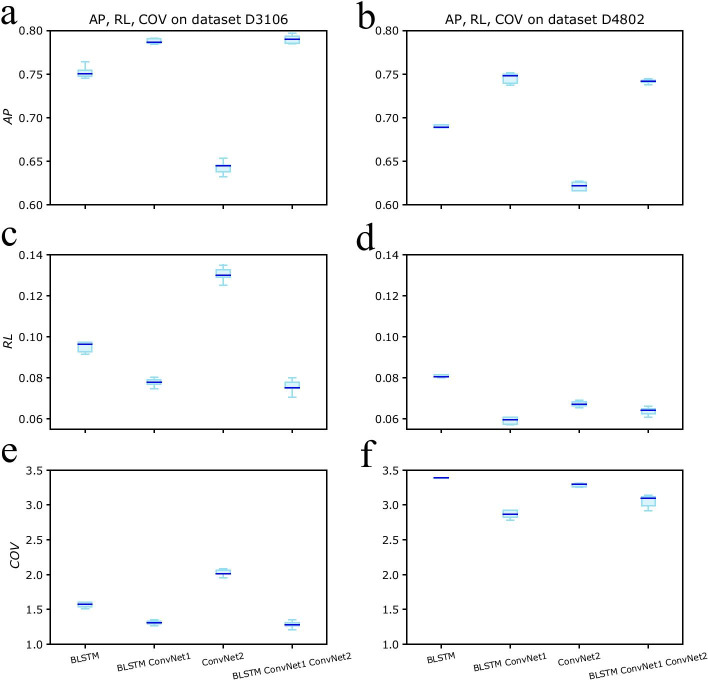
Box chart of prediction accuracy on benchmark datasets* D*3106 and* D*4802. The three plots in left column are box charts of average precision (AP), ranking loss (RL), coverage (COV) on dataset* D*3106, and the plots in right column are box charts of corresponding results on dataset* D*4802. Model BLSTM + ConvNet1 + ConvNet2 is the best one among the four models with highest average precision and smallest divergence on dataset* D*4802. However, the ranking loss and coverage of this model is greater than the ones of model BLSTM + ConvNet1. On dataset* D*3106, model BLSTM + ConvNet1 + ConvNet2 is better than the other three models in average precision, ranking loss and coverage. In average precision and coverage, those four models perform better on dataset* D*3106 than on dataset* D*4802. The ranking loss values of those four models on dataset* D*4802 are lower than the values on dataset* D*3106.** a** This plot shows the average precisions of model BLSTM, model BLSTM + ConvNet1, model ConvNet2 and model BLSTM + ConvNet1 + ConvNet2 when they are tested on dataset* D*3106 with five-fold cross validation. Model BLSTM + ConvNet1 + ConvNet2 has the greatest average precision than the other three models, while model BLSTM + ConvNet1 has stable performance.** b** The average precisions of those four models when they are tested on dataset** D**4802. Model BLSTM + ConvNet1 has the best performance on this dataset, however model BLSTM + ConvNet1 + ConvNet2 is more stable on this dataset.** c** The ranking loss values of those four models when they are tested on dataset* D*3106.** d** The ranking loss of them with dataset* D*4802.** e** The coverage results of predictions by model BLSTM, model BLSTM + ConvNet1, model ConvNet2 and model BLSTM + ConvNet1 + ConvNet2 on dataset* D*3106.** f** The coverage results of predictions by those four models on dataset* D*4802

When evaluating the performance of the four models as multi-label classification models, we can get similar conclusion from the results of ranking loss, coverage and average precision. The results of experiment are listed in Table 4. The model ConvNet2 has the worst performance on both dataset D3106 and dataset D4802. And the best one is the joined model with average precision of 0.7901, ranking loss of 0.0758 and coverage of 1.2848 on dataset D3106. The prediction accuracy of the joined model on dataset D4802 is not as good as the accuracy on dataset D3106, but the model can still get average precision of 0.7414 and ranking loss of 0.0637. We use fivefold cross validation to test our method comprehensively and plot the box chart of accuracy in Figure 4. Additional file 1: Table S4 contains ranking loss, coverage and average precision of fivefold cross validation on dataset *D*3106 and *D*4802. All the four models were tested on dataset *D*3106 and *D*4802 and the corresponding validation results are in Additional file 1: Table S4.

**Table 5:** The average precision, ranking loss and coverage of IMMMLGP, Hum-mPloc, mGOF-loc, MKSVM, FSVM-KNR and out method when they are tested on datasets *D*3106 and *D*4802

	***D*3106**			***D*4802**		
	***RL***	***Cov***	***AP***	***RL***	***Cov***	***AP***
IMMMLGP	0.4190	4.3030	0.5810	0.2436	4.9772	0.5725
Hum-mPloc	0.4906	5.3170	0.5790	0.3145	5.6830	0.5644
mGOF-loc	–	–	–	**0.0606**	3.0227	0.6482
MKSVM	0.1085	1.7193	0.7065	0.0662	2.9753	0.6889
FSVM-KNR	0.1071	1.7025	0.7108	0.0971	**2.6339**	0.6916
Our Method	**0.0758**	**1.2848**	**0.7901**	0.0637	3.0528	**0.7414**

Our method has great improvements on subcellular localization prediction than five currently available methods when tested on dataset *D*3106. The average precision of our method is 0.7901 which is 0.08 greater than the average precision of FSVM-KNR. The ranking loss and coverage of our method are lower than the values of other five methods. On dataset *D*4802, our method did not get the lowest ranking loss and coverage. However, the average precision of our method on dataset *D*4802 is the highest among those six methods with 0.7414. The best values are listed out with bold text

## Discussion

The experiment results listed in Additional file 1: Table S4 show that model BLSTM plus 2 ConvNet has highest accuracy on dataset *D*3106 and model BLSTM plus ConvNet1 is the best with dataset *D*4802. According to average precision and coverage results, model BLSTM plus 2 ConvNet performs better on dataset *D*3106 than on dataset *D*4802, but the ranking loss value of it on *D*4802 is lower than the value on *D*4802. The possible reason of bad performance on dataset *D*4802 is that the unbalanced samples takes higher proportion in *D*4802 which makes model over-fitting under same training procedure. Samples which have high correlation between subcellular locations are suitable to our method. Since our method considers relations between each subcellular location as a feature, high correlation improves the prediction accuracy of subcellular location, of which the training samples are insufficient. Our method takes advantage of this relation and the accuracy of prediction has significant improvement.

The t test statistic of average precision, ranking loss and coverage is in Additional file 1: Table S5. In order to make t test statistic on prediction result, the prediction task was taken out on sub-group testing samples multiple times. The detailed information about average precision, ranking loss and coverage on dataset *D*3106 and *D*4802 is listed in Additional files 6 and 7 respectively. The data in Additional file 1: Table S5 shows that ConvNet2 has nothing to do with average precision when the method was tested on dataset *D*3106 as the change between average precision values is insignificant ($p_{value} > 0.5$). The differences of ranking loss and coverage are significant. With ConvNet2, our method gets better ranking loss and coverage on dataset *D*3106 with significant decrease ($p_{value} < 0.5$). However, on dataset *D*4802, ConvNet2 makes the situation worse. The reason caused this situation could be the limitation of our method on datasets which has less multi-cellular samples. From Additional file 1: Figure S3 we can see that dataset *D*4802 has more 2-cellular samples than dataset *D*3106 while 3-cellular samples and 4-cellular samples in *D*4802 are less than the ones in *D*3106. The percentages of 3-cellular samples and 4-cellular samples in dataset *D*3106 are 8.17% and 0.57% respectively, while the percentages of those kind of samples are 2.96% and 0.22% in dataset *D*4802. Since *D*4802 covers total of 33 subcellular locations and *D*3106 covers only 14 subcellular locations, the cellular position density in *D*4802 is lower than the one in *D*3106. Then ConvNet2 reduces ranking loss and coverage when the dataset contains more multi-cellular samples and the density of cellular coverage is higher. However, this conclusion needs more evaluation of those models on different datasets, which could be done in future researches.

We compared our method with five currently available methods which had been tested on the benchmark datasets D3106 and D4802. The evaluation results are listed in Table 5. The results in this table show that our method performs better on protein subcellular localization prediction than the other five methods. Our method gets average precision 0.7901 which is 0.08 higher than the average precision of method FSVM-KNR on dataset D3106, and both the ranking loss and coverage are also better than method FSVM-KNR. Only the coverage of our method on D4802 is worse than the coverage of FSVM-KNR. The t test statistic results of average precision are listed in Additional file 1: Table S6. The results in this table show that our method has higher average precision than the other four prediction methods on dataset *D*3106 and *D*4802. The improvements on average precision by our method are significant.

Additional file 1: Table S7 is the t test statistic results of ranking loss. On dataset *D*3106, our method is better than the other four methods with great significance. Model BLSTM plus ConvNet1 is also better than the other four methods. On dataset *D*4802, our method has no big advantage than other four methods even it performs better than methods IMMMLGP, Hum-mPloc and FSVM-KNR. Our method only got similar ranking loss values with method MKSVM since the difference between them is insignificant ($p_{value} > 0.5$). When compared with method mGOF-loc, our method is worse than mGOF-loc. The ranking loss of our method increases to 0.0637 with a difference of 0.0031 from ranking loss of mGOF-loc and t test shows that this difference is significant ($p_{value} < 0.05$). The t test statistic results of coverage, which are listed in Additional file 1: Table S8, deliver the same conclusion as the statistic results of ranking loss. Our method outperforms methods IMMMLGP, Hum-mPloc, MKSVM and FSVM-KNR on dataset *D*3106 with significant decrease in coverage values ($p_{value} < 0.05$). However our method is only better than methods IMMMLGP, Hum-mPloc on dataset *D*4802. It has similar coverage as methods MKSVM and mGOF-loc with insignificant differences ($p_{value} > 0.05$), but it is worse than method FSVM-KNR by an increase of 0.4 on coverage. So, from those results, we can see that our method performs well on *D*3106 while it does not have good performance on *D*4802, which means it has limitations on some kinds of datasets, such as datasets have few multi-cellular samples and datasets have low cellular density. Previous research shows that almost half of human proteins localize to multiple subcellular locations [73], so our method has potential to perform well at real biological problems.

## Conclusion

The experiment results in the previous section show that bidirectional LSTM and ConvNet are able to accurately predict protein subcellular localization with information of protein sequences and evolution matrices. By comparing our method with five currently available methods, it is obvious that our method outperforms those five. Therefore, the method we proposed is excellent in protein subcellular localization prediction. With the prediction results of our method, the locations and functions of proteins can be refined to remove bias or errors existed in traditional methods. Also, predictions at different stage can find out the changes of protein locations, which can be used to make biological pathway analysis. At the same time, we also realize that our method performs worse on some datasets due to the composition of training samples. For example, the performance on dataset *D*4802 is not as good as the performance on dataset *D*3106 because the unbalance property of samples and the high correlations between subcellular locations.

Furthermore, our experiments show that evolution information alone is insufficient to classify the proteins into groups, and amino acid composition of proteins is crucial in the prediction of protein subcellular localization. However, it is remain reasonable to apply evolution information in biological prediction problems as an additional information, since our method which applies evolution information achieves small improvements on prediction accuracy of multi-cellular localization, especially when a dataset has a large proportion of multi-cellular samples. Besides PSSM, there are also other forms of evolution information such as mutation rate, mutation bias, phylogenetic tree, and so on. Those kinds of information are also helpful to biological predictions.

Sequence information encoding is very important to the success of biological prediction methods. The performance of model BLSTM shows that protein sequence encoding method is essential in deep learning-based protein subcellular localization prediction methods. There is an increasing number of deep learning models that are being proposed to process sequence data, which are beneficial to protein classification. However, a fine encoding method is pivotal in translating raw protein sequences into learn-able matrices in order to utilize the proposed models in bioinformatics research.

Our method can not only predict subcellular locations of proteins, but also can predict other properties of proteins based on protein sequence and evolution information. For example, our method can predict protein functions besides locations. Novel application such as protein structure prediction can be developed based on the architecture of our model. However, this needs further work to make it work.

## Supplementary Information

**Additional file 1**. This file contains detailed information about the performance of this method when it is trained and tested on dataset D3106 and D4802.

**Additional file 2**. This file contains additional information about dataset D3106, correlation between each pair of subcellular locations in dataset D3106.

**Additional file 3**. This file contains additional information about dataset D4802, correlation between each pair of subcellular locations in this dataset.

**Additional file 4**. This file lists the AUC values of this method on dataset D3106.

**Additional file 5**. This file lists the AUC values of this method on dataset D4802.

**Additional file 6**. The average precision and ranking loss of this mothod when tested on dataset D3106.

**Additional file 7**. The average precision and ranking loss of this method when tested on dataset D4802.

## Data Availability

The datasets used and analysed during the current study are available in the figshare repository, https://doi.org/10.6084/m9.figshare.14378630.v1. The python code described in this manuscript are available in the github reposiroty, https://github.com/Paeans/subcellular.

## Abbreviations

AP: Average precision
AUC: Area under the ROC curve
BLAST: Basic local alignment search tool
BLSTM: Bidirectional long short-term memory network
ConvNet: Convolutional neural network
Cov: Coverage
FSVM: Fuzzy support vector machine
GO: Gene ontology
GP: Gaussian process
KNR: Kernelized neighborhood representation
LASSO: Least absolute shrinkage and selection operator
lnc-RNA: Long non-coding RNA
LSTM: Long short-term memory network
MCC: Matthews correlation coefficient
PPV: Positive predictive value
PsePP: Pseudo-physicochemical properties
PsePSSM: Pseudo-position specific scoring matrix
PSI-BLAST: Position specific iterated BLAST
PSSM: Position specific scoring matrix
ReLU: Rectified linear activation function
RNN: Recurrent neural network
RL: Ranking loss
ROC: Receiver operating characteristic curve
SVM: Support vector machine
TPR: True positive rate
