# Supplementary material for: Predicting subcellular location of protein with evolution information and sequence-based deep learning
Source: BMC Bioinformatics. 2021 Oct 22;22(Suppl 10):515. doi: 10.1186/s12859-021-04404-0 (PMC8539821; doi:10.1186/s12859-021-04404-0)
Supplement: Supplementary file 1 — Additional file 1. This file contains detailed information about the performance of this method when it is trained and tested on dataset D3106 and D4802. [file 12859_2021_4404_MOESM1_ESM.pdf]

Predicting subcellular location of protein with evolution information and sequence-based deep learning - Supplementary Tables and Figures

Zhijun Liao, Gaofeng Pan, Chao Sun and Jijun Tang

Table S1: Statistic of each subcellular location prediction AUC on dataset D3106

	5-Fold Cross Validation AUC											
**D3106**	BLSTM			BLSTM + ConvNet1			ConvNet2			BLSTM + ConvNet1 + ConvNet2		
	MAX	AVG	STD	MAX	AVG	STD	MAX	AVG	STD	MAX	AVG	STD
Nucleus	.8769	.8710	.0056	.8973	.8962	.0015	.7695	.7611	.0071	**.9001**	***.8982***	*.0013*
Centrosome	.9135	.8992	.0094	.9368	***.9302***	*.0052*	.8309	.8222	.0112	**.9379**	.9282	.0092
Endosome	.9018	.8594	.0374	**.9230**	.8801	.0233	.7622	.7104	.0277	.9002	***.8838***	*.0171*
Lysosome	.9242	.9059	*.0097*	.9381	.9129	.0176	.8443	.8244	.0191	**.9458**	***.9239***	.0263
Microsome	.9081	.8817	.0193	**.9257**	***.9040***	.0249	.8714	.8246	.0320	.9157	.8924	*.0173*
Peroxisome	.8480	.8362	*.0096*	.9134	.8728	.0246	.7788	.7555	.0228	**.9285**	***.8954***	.0204
Cytoskeleton	.9078	.8847	.0152	**.9302**	***.9163***	*.0080*	.8050	.7812	.0163	.9203	.9084	.0091
Cytoplasm	.8501	.8414	.0072	.8834	.8807	*.0021*	.6601	.6473	.0111	**.8880**	***.8815***	.0053
Plasma Membrane	.8927	.8834	.0120	.9142	.9097	.0036	.7845	.7832	*.0012*	**.9148**	***.9101***	.0041
Endoplasmic Reticulum	.8889	.8644	.0192	.8972	.8921	*.0049*	.7262	.7055	.0131	**.9050**	***.8939***	.0071
Extracellular	.9057	.8974	.0061	.9225	.9124	*.0060*	.8785	.8526	.0177	**.9332**	***.9235***	.0063
Golgi Apparatus	.8706	.8575	*.0068*	**.9060**	***.8928***	.0119	.7058	.6934	.0084	.9013	.8892	.0110
Mitochondrion	.8894	.8805	.0100	**.9228**	.9055	.0132	.8391	.8103	.0153	.9199	***.9113***	*.0052*
Synapse	.8997	.8630	.0260	**.9379**	***.9088***	.0247	.7551	.7261	*.0155*	.8861	.8749	.0166

Table S2: Statistic of each subcellular location prediction AUC on dataset D4802

**D4802**		5-Fold Cross Validation AUC											
		BLSTM			BLSTM + ConvNet1			ConvNet2			BLSTM + ConvNet1 + ConvNet2		
		MAX	AVG	STD	MAX	AVG	STD	MAX	AVG	STD	MAX	AVG	STD
Membrane	Apical Plasma	.8368	.7966	.0389	**.9297**	.8852	.0467	.7988	.7755	*.0209*	.9189	***.8937***	.0284
	Basolateral Plasma	.8714	.8445	.0209	.9260	.9017	*.0195*	.8134	.7628	.0324	**.9434**	***.9123***	.0437
	Inner Mitoch	.8861	.7537	.1208	**.9892**	.8703	.0612	.7969	.6788	.0770	.9691	***.9121***	*.0546*
	Plasma	.8804	.8713	.0064	**.9064**	***.8991***	.0044	.8057	.7990	.0039	.9031	.8977	*.0035*
Cellular	Component	.8915	.7223	.1449	**.9552**	***.8749***	*.0680*	.8560	.6950	.1329	.9411	.8321	.0719
	Extra	.8997	.8908	*.0066*	**.9159**	***.9017***	.0136	.8498	.8360	.0074	.9142	.9008	.0108
	Centrosome	.8838	.8788	*.0034*	**.9364**	***.9244***	.0080	.8359	.8170	.0110	.9202	.9063	.0137
	Lysosomes	.8896	.8251	.0373	.9047	***.8955***	*.0088*	.7620	.7428	.0176	**.9112**	.8899	.0137
	Melanosome	.7934	.6855	.0609	**.9451**	.8745	.0403	.7856	.7059	.0536	.9136	***.8769***	*.0259*
	Peroxisome	.8455	.7976	.0406	**.9168**	***.8903***	*.0160*	.7866	.7567	.0193	.9082	.8744	.0250
Cytoplas	Cytoplasm	.8327	.8211	.0079	.8626	***.8599***	*.0025*	.6617	.6579	.0031	**.8642**	.8582	.0041
	Vesicles	.8001	.7732	*.0142*	**.9191**	***.8771***	.0250	.6811	.6445	.0374	.9123	.8746	.0231
Endosomes	Early	.8580	.8008	.0317	.8927	***.8712***	.0144	.7427	.7262	*.0115*	**.9123**	.8674	.0306
	Endosome	.8193	.7884	.0201	.8739	***.8655***	.0059	.6566	.6512	*.0048*	**.8788**	.8638	.0097
	Late	.8686	.8212	.0414	.9425	***.9027***	*.0351*	.7881	.6895	.0645	**.9527**	.8950	.0382
Golgi	Apparatus	.8454	.8221	.0144	**.8849**	***.8786***	.0051	.6612	.6547	*.0044*	.8777	.8721	.0049
	Cis Cisterna	.8602	.7777	.0935	.9158	.8124	.0749	.7196	.6451	*.0505*	**.9604**	***.8275***	.0945
	Trans Cisterna	.8606	.7217	.1470	**.9952**	.8138	*.1028*	.7530	.5752	.2000	.9810	***.8464***	.1413
	Trans Face	.9001	.8710	.0243	**.9477**	***.9194***	.0263	.6895	.6348	.0423	.8969	.8816	*.0155*
	Medial	.9279	.8269	.0564	**.9620**	.8820	.0543	.6406	.5460	.0794	.9591	***.9045***	*.0418*
Nu	Envelope	.8161	.7694	.0409	**.8880**	***.8729***	.0123	.6254	.6147	*.0107*	.8872	.8616	.0170

**D4802**		5-Fold Cross Validation AUC											
		BLSTM			BLSTM + ConvNet1			ConvNet2			BLSTM + ConvNet1 + ConvNet2		
		MAX	AVG	STD	MAX	AVG	STD	MAX	AVG	STD	MAX	AVG	STD
	Nucleolus	.8643	.8502	.0075	**.8896**	***.8789***	*.0069*	.7522	.7435	.0072	.8875	.8754	.0080
	Nucleus	.8809	.8747	.0039	**.9019**	***.8996***	*.0020*	.7770	.7718	.0063	.9016	.8939	.0041
	Secretory Granule	.8470	.6958	.1141	**.9288**	.7595	.1484	.5080	.4683	.0406	.8061	***.7887***	*.0156*
Vesicles	Secretory	.8290	.6371	.1478	**.9303**	***.8590***	*.0556*	.8719	.5394	.2170	.9015	.7830	.1238
	Synaptic	.7072	.6592	.0277	**.9281**	***.8833***	.0244	.6693	.6530	*.0132*	.8620	.8430	.0254
	Transport	.8589	.7650	*.0889*	**.9551**	***.7916***	.0938	.7800	.5962	.1642	.8927	.6708	.1919
	Tight Junction	**.9092**	.7779	.0681	.8940	***.8768***	.0222	.7519	.7008	.0479	.8746	.8554	*.0163*
	Cytoskeleton	.8580	.8250	.0190	**.9157**	***.8876***	.0197	.7581	.7443	*.0077*	.8940	.8824	.0099
	ERGIC	.8830	.7429	.0844	.9650	***.8969***	.0623	.7041	.6431	*.0458*	**.9792**	.8389	.0788
	Endoplasmic Reticulum	.7767	.7504	.0186	**.8878**	***.8604***	.0174	.6253	.6081	*.0119*	.8681	.8547	.0149
	Microtubule	.8069	.7595	.0451	**.9298**	.8714	.0330	.8047	.7765	.0194	.9271	***.9003***	*.0181*
	Mitochondria	.8704	.8573	.0098	**.9077**	.8955	*.0064*	.8114	.7998	.0126	.9043	***.8964***	.0076

Table S3: t-test of AUC on 4 models with datasets D3106 and D4802

		BLSTM		BLSTM + ConvNet1		ConvNet2	
		t-value	p-value	t-value	p-value	t-value	p-value
D3106	BLSTM + ConvNet1	6.8341	2.43e-10	/		/	
	ConvNet2	13.6827	1.82e-27	17.5856	4.71e-37		
	BLSTM + ConvNet1 + ConvNet2	6.9257	1.50e-10	**0.0027**	**9.98e-01**	17.6485	3.34e-37
D4802	BLSTM + ConvNet1	9.9172	1.90e-20	/		/	
	ConvNet2	9.1517	6.12e-18	19.0119	7.43e-55		
	BLSTM + ConvNet1 + ConvNet2	8.1969	5.61e-15	**1.2428**	**2.15e-01**	17.0774	3.15e-47

Table S4: Average precision (AP), Ranking loss (RL) and Coverage (Cov) of 4 models. Models are tested on datasets D3106 and D4802 with 5-fold cross validation. And all the five-fold test results and their corresponding average values are listed.

			5-Fold Cross Validation					Average
D3106	BLSTM	RL	0.0965	0.0916	0.0973	0.1052	0.0928	0.0967
		Cov	1.5740	1.5097	1.6049	1.7229	1.5359	1.5895
		AP	0.7476	0.7640	0.7503	0.7452	0.7546	0.7523
	BLSTM + ConvNet1	RL	0.0780	0.0804	0.0769	0.0792	0.0747	0.0778
		Cov	1.3342	1.3532	1.2969	1.3087	1.2636	1.3113
		AP	0.7910	0.7844	0.7853	0.7867	0.7906	0.7876
	ConvNet2	RL	0.1341	0.1253	0.1272	0.1301	0.1307	0.1294
		Cov	2.0872	1.9539	2.0139	2.0138	2.0621	2.0262
		AP	0.6324	0.6536	0.6452	0.6449	0.6376	0.6430
	BLSTM + ConvNet1 + ConvNet2	RL	0.0749	0.0751	0.0706	0.0785	0.0801	**0.0758**
		Cov	1.2681	1.2115	1.3496	1.2824	1.3124	**1.2848**
		AP	0.7903	0.7932	0.7967	0.7853	0.7849	**0.7901**
D4802	BLSTM	RL	0.0805	0.0816	0.0870	0.0802	0.0806	0.0820
		Cov	3.4141	3.3900	3.3904	3.3864	3.3772	3.3916
		AP	0.6890	0.6916	0.6823	0.6993	0.6883	0.6901
	BLSTM + ConvNet1	RL	0.0609	0.0666	0.0571	0.0574	0.0595	**0.0603**
		Cov	2.9260	3.2157	2.7791	2.8273	2.8645	**2.9225**
		AP	0.7395	0.7372	0.7514	0.7499	0.7484	**0.7453**
	ConvNet2	RL	0.0692	0.0665	0.0671	0.0683	0.0655	0.0673
		Cov	3.3119	3.2634	3.2571	3.3015	3.3002	3.2868
		AP	0.6161	0.6216	0.6260	0.6161	0.6270	0.6214
	BLSTM + ConvNet1 + ConvNet2	RL	0.0641	0.0624	0.0649	0.0609	0.0662	0.0637
		Cov	3.0970	2.9896	3.1361	2.9197	3.1217	3.0528
		AP	0.7377	0.7406	0.7416	0.7428	0.7445	0.7414

Table S5: t-test of Average precision (AP), Ranking loss (RL) and Coverage (COV) on 4 models with datasets D3106 and D4802.

			BLSTM		BLSTM + ConvNet1		ConvNet2	
			t-value	p-value	t-value	p-value	t-value	p-value
D3106	BLSTM + ConvNet1	AP	24.3248	2.16e-61	/		/	
		RL	22.0541	3.17e-55				
		COV	22.2753	7.74e-56				
	ConvNet2	AP	63.3824	1.88e-133	90.0520	1.26e-162	/	
		RL	38.0276	5.94e-93	64.9183	2.04e-135		
		COV	34.4171	1.69e-85	63.5249	1.23e-133		
	BLSTM + ConvNet1 + ConvNet2	AP	24.0411	1.23e-60	**1.9464**	**5.30e-002**	85.6818	1.87e-158
		RL	23.0946	4.38e-58	2.6199	0.95e-002	63.4763	1.42e-133
		COV	22.8474	2.07e-57	2.2012	2.89e-002	61.4233	6.99e-131
D4802	BLSTM + ConvNet1	AP	36.1469	3.88e-89	/		/	
		RL	24.2200	4.12e-61				
		COV	25.7797	3.42e-65				
	ConvNet2	AP	40.8202	2.32e-98	83.5651	2.32e-156	/	
		RL	15.0811	1.00e-34	15.2605	2.84e-035		
		COV	15.0034	1.74e-34	17.0647	9.43e-041		
	BLSTM + ConvNet1 + ConvNet2	AP	27.8925	1.65e-70	6.9783	4.39e-011	71.9737	6.28e-144
		RL	18.7163	1.14e-45	6.5072	6.13e-010	7.3027	6.72e-012
		COV	20.4098	1.38e-50	6.2787	2.12e-009	9.3781	1.59e-017

Table S6: t-test of Average precision (AP) on our method and other five methods. The tested methods are IMMMLGP, Hum-mPloc, MKSVM, FSVM-KNR and mGOF-loc. The datasets used to test are D3106 and D4802.

**AP**		BLSTM		BLSTM + ConvNet1		ConvNet2		BLSTM + ConvNet1 + ConvNet2	
		t-value	p-value	t-value	p-value	t-value	p-value	t-value	p-value
D3106	IMMMLGP	51.80	8.31e-07	152.45	1.11e-08	17.07	6.91e-05	92.02	8.36e-08
	Hum-mPloc	52.41	7.93e-07	153.92	1.07e-08	17.62	6.09e-05	92.90	8.05e-08
	MKSVM	13.86	1.57e-04	59.85	4.67e-07	-17.61	6.11e-05	36.78	3.26e-06
	FSVM-KNR	12.56	2.31e-04	56.68	5.80e-07	-18.80	4.72e-05	34.89	4.03e-06
	mGOF-loc	/							
D4802	IMMMLGP	28.09	9.55e-06	91.41	8.58e-08	43.11	1.73e-06	150.11	1.18e-08
	Hum-mPloc	30.03	7.32e-06	95.56	7.19e-08	50.60	9.13e-07	157.30	9.79e-09
	MKSVM	0.17	**8.75e-01**	31.77	5.85e-06	-64.43	3.47e-07	46.68	1.25e-06
	FSVM-KNR	-0.48	**6.56e-01**	30.38	6.98e-06	-66.92	2.99e-07	44.28	1.55e-06
	mGOF-loc	9.93	5.77e-04	52.62	7.81e-07	-26.83	1.15e-05	82.85	1.27e-07

Table S7: t-test of Ranking loss (RL) on our method and other five methods. The tested methods are IMMMLGP, Hum-mPloc, MKSVM, FSVM-KNR and mGOF-loc. The datasets used to test are D3106 and D4802.

**RL**		BLSTM		BLSTM + ConvNet1		ConvNet2		BLSTM + ConvNet1 + ConvNet2	
		t-value	p-value	t-value	p-value	t-value	p-value	t-value	p-value
D3106	IMMMLGP	134.49	1.83e-08	348.54	4.07e-10	174.64	6.45e-09	214.23	2.85e-09
	Hum-mPloc	164.37	8.22e-09	421.70	1.90e-10	217.98	2.66e-09	258.92	1.33e-09
	MKSVM	4.92	7.92e-03	31.30	6.21e-06	-13.34	1.83e-04	20.42	3.39e-05
	FSVM-KNR	4.34	1.23e-02	29.87	7.48e-06	-14.19	1.43e-04	19.55	4.04e-05
	mGOF-loc	/							
D4802	IMMMLGP	62.21	4.00e-07	156.25	1.01e-08	431.96	1.72e-10	195.55	4.10e-09
	Hum-mPloc	89.28	9.44e-08	216.43	2.73e-09	606.64	4.43e-11	272.63	1.09e-09
	MKSVM	-5.50	5.32e-03	5.67	4.78e-03	-5.11	6.92e-03	2.68	**5.52e-02**
	FSVM-KNR	6.29	3.26e-03	31.90	5.76e-06	71.02	2.36e-07	36.27	3.45e-06
	mGOF-loc	-7.64	1.58e-03	0.92	**4.12e-01**	-18.91	4.61e-05	-3.41	2.71e-02

Table S8: t-test of Coverage (COV) on our method and other five methods. The tested methods are IMMMLGP, Hum-mPloc, MKSVM, FSVM-KNR and mGOF-loc. The datasets used to test are D3106 and D4802.

COV		BLSTM		BLSTM + ConvNet1		ConvNet2		BLSTM + ConvNet1 + ConvNet2	
		t-value	p-value	t-value	p-value	t-value	p-value	t-value	p-value
D3106	IMMMLGP	73.16	2.09e-07	193.74	4.26e-09	99.15	6.20e-08	131.01	2.04e-08
	Hum-mPloc	100.50	5.88e-08	259.41	1.32e-09	143.30	1.42e-08	175.02	6.39e-09
	MKSVM	3.50	2.49e-02	26.42	1.22e-05	-13.36	1.81e-04	18.86	4.66e-05
	FSVM-KNR	3.05	3.82e-02	25.33	1.44e-05	-14.10	1.47e-04	18.13	5.44e-05
	mGOF-loc	/							
D4802	IMMMLGP	9.62	6.52e-04	41.21	2.07e-06	124.13	2.53e-08	45.77	1.36e-06
	Hum-mPloc	15.60	9.86e-05	55.12	6.49e-07	176.59	6.17e-09	62.56	3.91e-07
	MKSVM	-7.34	1.84e-03	1.77	**1.52e-01**	-24.66	1.60e-05	-1.84	**1.39e-01**
	FSVM-KNR	-10.23	5.15e-04	-4.96	7.70e-03	-50.04	9.54e-07	-9.96	5.70e-04
	mGOF-loc	-6.93	2.27e-03	2.70	**5.41e-02**	-21.14	2.96e-05	-0.72	**5.13e-01**

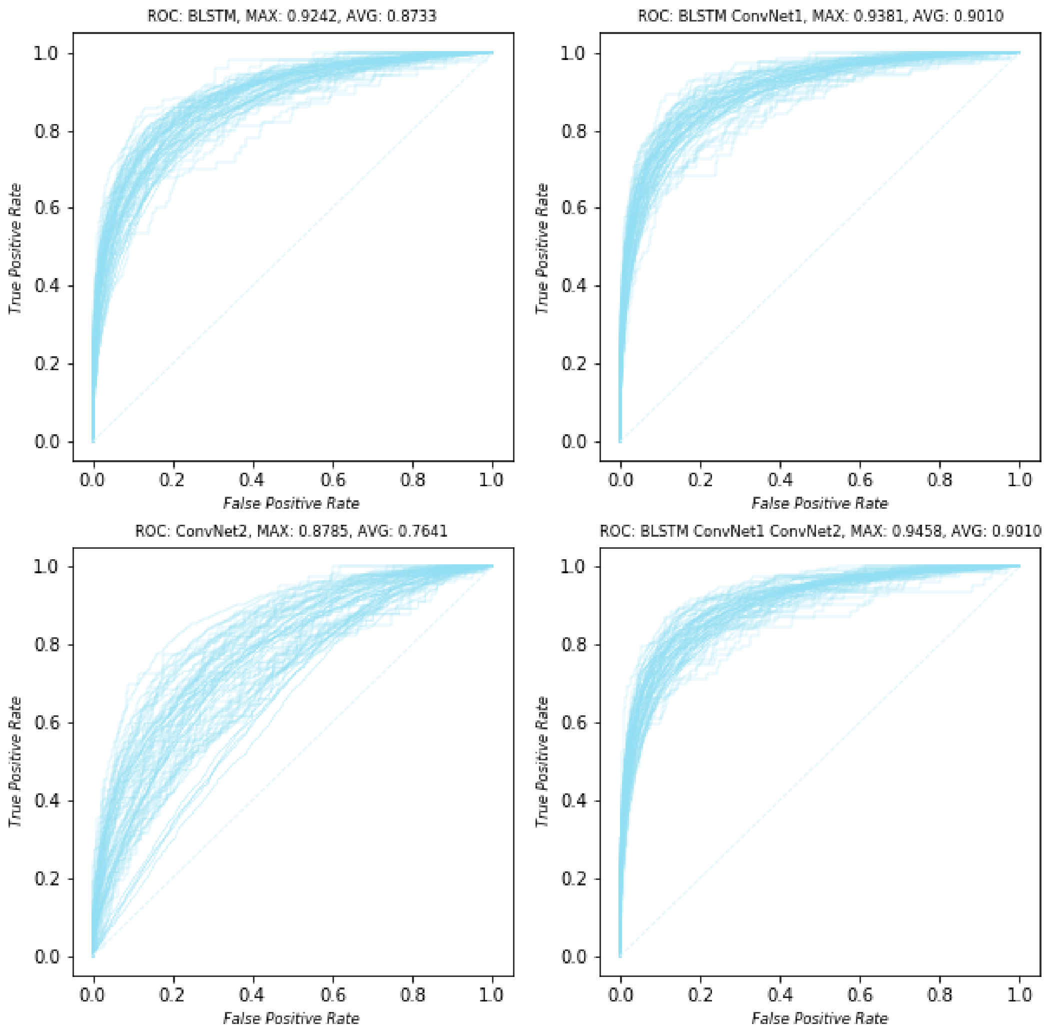

Figure S1: ROC curves (Receiver Operating Characteristic) of model BLSTM, BLSTM + ConvNet1, ConvNet2 and BLSTM + ConvNet1 + ConvNet2 on dataset D3106 with 5-fold cross validation. All the 5-fold testing results are plotted. Each curve represents a prediction of a subcellular location.

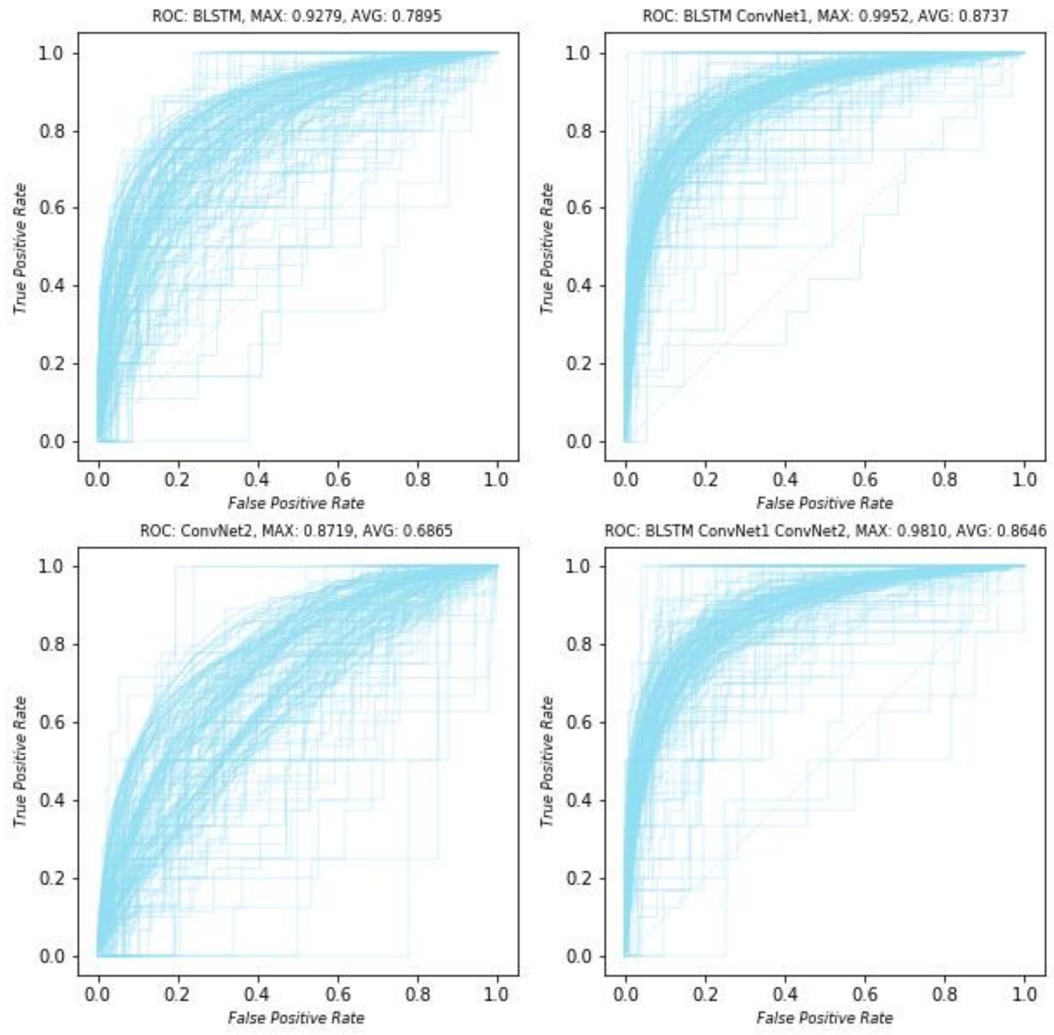

Figure S2: ROC curves (Receiver Operating Characteristic) of model BLSTM, BLSTM + ConvNet1, ConvNet2 and BLSTM + ConvNet1 + ConvNet2 on dataset D4802 with 5-fold cross validation. All the 5-fold testing results are plotted. Each curve represents a prediction of a subcellular location.

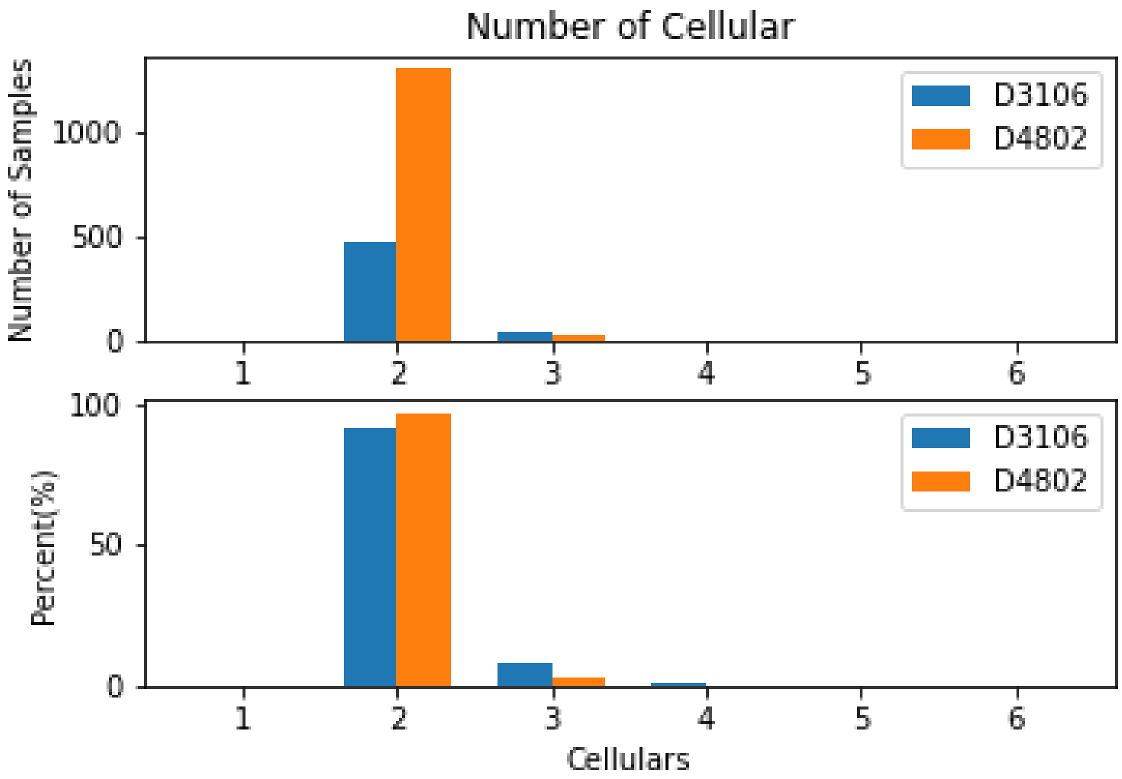

Figure S3: Number of multi-cellular samples in dataset D3106 and dataset D4802. In dataset D3106, there are more 3-cellular samples and 4-cellular samples. The percentages of 3-cellular samples and 4-cellular samples are higher in dataset D3106 than the proportions in dataset D4802.
